# The journey to a learning health system in primary care: a qualitative case study utilising an embedded research approach

**DOI:** 10.1186/s12875-022-01955-w

**Published:** 2023-01-19

**Authors:** Genevieve Dammery, Louise A. Ellis, Kate Churruca, Janani Mahadeva, Francisco Lopez, Ann Carrigan, Nicole Halim, Simon Willcock, Jeffrey Braithwaite

**Affiliations:** 1 Centre for Healthcare Resilience and Implementation Science, Australian Institute of Health Innovation, Macquarie University, North Ryde, Sydney, NSW 2109 Australia; 2 NHMRC Partnership Centre for Health System Sustainability, Australian Institute of Health Innovation, Macquarie University, Sydney, NSW Australia; 3 MQ Health, Macquarie University, Sydney, NSW Australia; 4 Australian Institute of Health Innovation, Macquarie University, Sydney, NSW Australia

**Keywords:** Learning health system, General practice, Primary care, Quadruple aim

## Abstract

**Background:**

Healthcare systems may be resilient and adaptive, but they are not fit for purpose in their current state. Increasing threats to health system sustainability have underscored the need to move towards a learning health system in which research and data are used routinely in clinical practice to facilitate system improvement. This study aimed to establish which elements of the learning health system were being realised within a university-based general practice and determine acceptability from staff to embrace further the transition towards a learning health system.

**Methods:**

Semi-structured interviews were conducted with practice staff, including clinical and administrative staff, to determine the current state of the learning health system in the practice. An embedded researcher was placed within the general practice on a part-time basis to investigate the learning health system model. Interviews were transcribed and thematically analysed based on the National Academy of Medicine’s framework of learning health systems.

**Results:**

In total, 32 (91%) practice staff were interviewed, comprising general practitioners (*n* = 15), nurses (*n* = 3), administrative staff (*n* = 13), and a psychologist (*n* = 1). Participants indicated that the practice was operating with several characteristics of a learning health system (e.g., emphasising science and informatics; focusing on patient-clinician partnerships; applying incentives; supporting a continuous learning culture; and establishing structures and governance for learning). These measures were supported by the university-based setting, and resultant culture of learning. Nevertheless, there were areas of the practice where the learning health system could be strengthened, specifically relating to the use of patient data and informatics. Staff generally expressed willingness to engage with the process of strengthening the learning health system within their practice.

**Conclusion:**

Although the idea of a learning health system has been gaining traction in recent years, there are comparatively few empirical studies presented in the literature. This research presents a case study of a general practice that is operating as a learning health system and highlights the utility of using the learning health system framework.

## Introduction

Health system performance has been described as the ‘60:30:10 Challenge’: 60% of care delivered is adherent to consensus-based guidelines; 30% of care is waste; and 10% results in direct harm to the patient, with these numbers remaining static for over three decades despite efforts to improve them [1]. Annually, healthcare accounts for 5% of the global carbon footprint, with this figure set to increase over the coming years [2]. The COVID-19 pandemic has emphasised how vulnerabilities within the health system can deeply affect human health and has revealed serious levels of burnout for professionals working in the system [3]. All these factors combined point to the need for robust and resilient healthcare systems globally; systems that are able to adapt to ongoing challenges and pressures. In response, the concept of a Learning Health System (LHS) was developed over several decades. According to the National Academy of Medicine (NAM; then the Institute of Medicine), an LHS is one where, “science, informatics, incentives and culture are aligned for continuous improvement and innovation” [4]. Core characteristics of an LHS include real time access to knowledge, patient-clinician partnerships, transparency on all aspects of care, and a leadership-instilled culture of learning [5]. Work in 2020 by Zurynski et al. added a fifth characteristic – structure and governance [5]. This is the modified NAM model (Table 1).

**Table 1:** LHS domains and characteristics identified by the modified NAM model [4, 5]

Domain	Characteristics	Description
**Science and Informatics**	*Real-time access to knowledge*	Best available evidence incorporated into clinical decision-making processes to improve the quality of care and patient safety.
	*Digital capture of the care experience*	Digital platforms (e.g., EHRs, disease registries, mobile devices) utilised for the real-time capture, production, and application of knowledge based on best available data.
**Patient-Clinician Partnerships**	*Engaged, empowered patients*	Patients, families, and caregivers are full partners in a patient-centred system.
**Incentives**	*Incentives aligned for value*	Policies actively encourage ongoing evaluation of care given and improvement of processes and support the provision of high-value care and reduction in wasteful practices. Incentives should be aligned across sectors, including health providers, health delivery systems, and patients, to provide better outcomes, improve efficiency, and increase engagement.
	*Full transparency*	All aspects of care, including safety, quality, processes, costs, and outcomes are recorded and available to stakeholders (patients, health professionals, managers) to improve patient care and decision making.
**Continuous Learning Culture**	*Leadership-instilled culture of learning*	Leaders instil a culture of collaboration and adaptability to support the learning process.
	*Supportive system competencies*	Staff training, skill building, and support to enable continuous refinement of processes and system improvements is implemented.
**Structure and Governance**		Policies, governance, and regulations aligned to facilitate research, collaboration, and learning.

Many existing LHSs described in the literature – whether aspiring or more fully fledged – are hospital-based and located in the United States [6]. Although there is some discussion of LHSs in relation to primary care, these generally centre on the role of primary care providers within the larger health system (e.g., Geisinger healthcare in the United States) [7], but not with the primary care provider being the main focus [8].

Primary care is typically the first contact that a patient has with the healthcare system [8]. In Australia alone, people engage with general practitioners (GPs) more than 150 million times annually [9], and the country’s primary care workforce consists of a range of medical and allied healthcare professionals [10]. In many countries, primary care providers are small businesses that operate independently, with the potential to make operationalising several core aspects of an LHS (i.e., linking data) difficult [8]. Whilst the quadruple aim - improving outcomes; lowering costs; and improving both the patient and clinician experience – is used to guide Quality Improvement (QI) in primary care globally [11], challenges arise when frontline workers (e.g., doctors, nurses) are required to implement change, due to their already heavy workloads and limited time to focus on QI or implementation activities [12]. As such, it is necessary to understand the degree to which an organisation is operating, for example as an LHS, before any further implementation is possible.

It has been estimated that almost 85% of medical research evidence does not enter clinical practice [13]. One strategy to address this challenge is to use an embedded researcher to support the evaluation or implementation of initiatives within healthcare settings. Using an embedded researcher, also known by other terms such as researcher-in-residence, is a mechanism to reduce the gap between researchers in the ivory tower or isolated laboratory and front line delivery of evidence-based medicine [14], with benefits of producing tailored research that uniquely responds to the context and culture of the setting that is being studied [15]. Where traditionally researchers are removed from the health system that they are studying or evaluating [16], embedded researchers are able to bridge this gap by working within the system that is being studied to the benefit of researchers and those working *in situ*.

### Study aims

The majority of literature discussing LHSs remains theoretical or normative in nature [17, 18], with an identified need for further empirical work specific to primary care [8]. Thus, the objective of this paper is to present an empirical case study of an LHS in primary care. The study had two overarching aims:

1. To understand the degree to which a large general practice is currently operating within an LHS framework and establish the acceptability of staff to further embrace and implement the LHS model;
2. To interrogate the validity of the embedded research model as a tool for understanding the setting, facilitating data collection and more broadly assisting with research partnerships, co-design, and QI.

## Methods

A co-designed, qualitative approach was undertaken involving academic researchers from the Australian Institute of Health Innovation (AIHI) and staff from MQ Health General Practice (MQGP). The core research team consisted of two senior academics (JB, LAE), a Research Fellow (KC), one Research Assistant who was partially embedded within the general practice (GD), three practicing academic General Practitioners (SW, SV, JM) and the Business Manager for MQGP (FL).

### Site

MQGP was selected as the clinical microsystem to partner with for this research due to its unique location, involvement in research and teaching activities, and strong engagement in QI initiatives. The general practice is a department of MQ Health, a university-owned not-for-profit health enterprise in Sydney, Australia. It operates across two sites: one situated adjacent to a hospital on a university campus, and the other in a suburban location. The practice, like many in Australia, works closely with its local Primary Health Network (PHN)—a government-initiated independent organisation that works with healthcare services to streamline care [19]. The practice has a strong focus on QI initiatives, with the ultimate goal of achieving the quadruple aim [11]. Most of the practice staff (with the exception of two GPs) are employees of MQ Health, and all have access to resources available to university employees.

### Embedded research approach and project timeline

A research assistant was embedded within the practice to conduct practice-level data collection and analysis, liaise frequently with the business manager and general practitioners, and work alongside the practice on multiple QI initiatives. The embedded researcher was included on all staff emails, attended the practice ‘strategy day’, and was introduced to practice staff at the GP clinic practice meeting. The ongoing research partnership between AIHI and MQGP began in April 2021, with the embedded research component commencing in July of 2021 and continuing until December 2021. Interviews were conducted in October 2021.

### Data collection

Semi-structured interviews were conducted with clinical and non-clinical practice staff to ensure that all staff perspectives were captured. Interview questions were designed by research staff using the modified NAM LHS framework [4, 5] as a guide. Questions were reviewed by multiple administrative and clinical staff at MQGP to ensure their accuracy and relevance to the practice. All practice staff were invited to take part and were provided with Participant Information and Consent Forms outlining the purpose of the research study prior to interview. Interviews were conducted by a senior research fellow or trained research assistant and were conducted in person at the general practice clinic, or via teleconference. Interviews were audio-recorded and transcribed verbatim.

### Analysis

Interview transcripts were imported into NVivo 20 [20] and analysed using a mix of deductive and inductive coding. A deductive approach guided by the modified NAM LHS framework and quadruple aim [11] was used to organise the data and understand the degree to which the practice operated as an LHS. Thematic coding was performed by GD, AC and NH, with input from LAE and KC. Regular meetings took place between the research team to ensure intercoder reliability.

In the presentation of results, extracts were edited minimally to improve readability without altering meaning. Staff were coded according to their roles (ADMIN: administrative staff, GP: general practitioner, NUR: nursing staff).

### Ethics

Ethics approval was granted by the Macquarie University Human Research Ethics Committee (ref no: 52021905624229). All participants provided full and informed written consent to take part in the research.

## Results

In total, 32 out of 35 (91%) practice staff were interviewed, comprising general practitioners (*n* = 15), practice nurses (*n* = 3), administrative staff (*n* = 13), and a psychologist (*n* = 1). Three clinicians were unable to attend their scheduled interview, and as data saturation was reached, these interviews were not rescheduled. Interviews lasted between 17 and 50 min (mean = 35.5). Participating staff had been working at MQGP for between three weeks and 15 years.

### Aim 1: MQ health general practice as a learning health system

#### Science and Informatics

An important element of an LHS is the use of digital platforms and EHRs. When asked about access to digital platforms to aid in their day-to-day work, several respondents highlighted the benefit of the practice’s affiliation with the university, which allowed for access to research and evidence through the university’s subscription to educational resources that may otherwise be inaccessible due to the associated high costs:

*“I’m lucky because I work at the University, so we do have access to [subscriptions], we have the Macquarie University ID, you can access that through the library … outside of this clinic it can get quite expensive.” (GP2)*

In addition to university-provided subscriptions, the local Primary Health Network (PHN) provided access to HealthPathways, an online primary care support tool [21], and CAT4, a clinical audit tool that gives practitioners an overview of their patient cohort as well as facilitating quarterly data transmission to the PHN to understand practice data in comparison to other practices in the same geographic location [22]. The CAT4 tool was able to extract data from the practice management and billing software and was accessible to all staff on request. Despite this, many GPs and administrative staff reported being unfamiliar with the software, and unaware of its utility. Of the GPs interviewed, nine had heard of the software, but only three had used it. Similarly, three of the administrative staff were familiar with the software, and only one had used it. On the other hand, all three nurses were aware of the software, with one having previously used it in the practice. Nevertheless, generally, practice staff expressed an interest in learning more about its utility:

*“I’ve had it shown to me, but I haven’t had to use it directly myself, so I know it conceptually, I think I could quite comfortably sit down and extract data and use it.” (GP10)*

*“As a practice we use it, I don’t necessarily do the extractions. [At] the practice I [previously worked] at I used to lead the accreditation, so I had become familiar with [CAT4 provider] and actually looking at things.” (GP3)*

Recently, the practice had also trialled an app to provide patients with access to their medical records and streamline the care process within the practice, as well as track referrals, prescriptions, and imaging results. The app was provided to patients at no cost and holds patient data for up to ten years.

*“We were looking at a way that patients can access the record and minimize the work that admin have to do and doctors have to do … that happens so often in our day, we are reprinting or re-emailing … that was one of the reasons for thinking about this app, because it is one app that does all of that.” (ADMIN12)*

Benefits of the app included simplified communication between patients, clinicians and the administrative team, and prevention of overlap in the work conducted. However, in real time, the app demonstrated limited use to inform clinical decision-making, instead serving as more of an administrative assistant tool and enabling digital record keeping for patients.

#### Patient-clinician partnerships

A key element and outcome of a successful LHS is patients who are empowered and engaged in their own care [4]. Practice staff were asked to describe current and future patient involvement within their practice. Staff outlined several ways that patients could be involved with the practice, with the most notable being a focus group, where patients were given the opportunity to provide feedback on the recently developed app for the practice:

*“We’ve been looking at an app called MyPractice so that the patient is more in control of their scripts, referrals results … We just weren’t sure how patients would feel about that, so we ran a patient focus group, and that went really well.” (GP12)*

Many staff members recognised the potential benefit of receiving regular, formal feedback from patients, whether in written survey format or via focus groups. However, there was mixed sentiment around how best to involve patients in the practice, considering issues surrounding patient recruitment and potential risk of bias:

*“You couldn’t take a random selection of patients. You have to be quite intentional about patients that you select. Some people don’t have much health literacy … you’re not going to get valuable feedback from someone who doesn’t really understand system to begin with.” (GP15)*

Recently, the practice had adopted a procedure in which all patients, following their appointment, were invited by email or text message to leave a review about their experiences visiting the clinic. Clinical staff commented on the benefits of these online reviews as a means of collecting patient feedback, connecting with patients and following up on patient concerns.

*“[A] patient made some comments on a Google review about how our booking system [has] been going. They identified some problems and [ADMIN1] saw this message, and he took action on it. I think he actually contacted the patient ask ‘what’s the problem’?” (NUR2)*

Furthermore, patient feedback was collected using the Practice Accreditation and Improvement Survey (PAIS) [23], a quality improvement tool recommended by the Royal Australian College of General Practitioners. This tool was distributed to patients on a biannual basis as part of the MQGP’s ongoing QI activities.

The involvement of patients in the practice was twofold: holding focus groups served to engage patients in the early stages of implementing new initiatives, whilst actively collecting feedback via online reviews gave patients a role in QI initiatives.

#### Incentives

Two important applications of incentives in an LHS model are: using incentives to reduce low-value care, as well as to assist with implementing changes within the organisation to stimulate its LHS journey. Financial incentives within the practice included key performance indicators (KPIs) and salaries for the doctors, often purported to increase value-based care instead of volume-based care. KPIs were awarded not only on the volume of care delivered, but also for engagement in teaching activities.

*“As part of our contract, we have KPIs. If you do meet your KPIs which are around your contributions to the practice, to education, to research, if you’re meeting all four or five criteria you’ll make a percentage on your billings, the gap between your threshold and your billings.” (GP1)*

As this model extended only to medical staff, some doctors made suggestions on how best to create an incentive system in the practice that benefits all staff and fosters collaboration instead of competition:

*“Part of the issue is getting the philosophy of what’s a proper incentive system … because it can then drive behaviours. You don’t want it to be competing with your colleagues, you want it to be collaborative and fair ... It also has to be inclusive, one of the discussions we’re having at the moment is why would you have incentives for the doctors and not the nurses and the administrative staff.” (GP10)*

Another characteristic of the LHS incentives domain is transparency. Securing suitable levels of transparency involves ensuring that care is continually improved among multiple dimensions (safety, quality, processes, costs and outcomes). Both administrative and clinical staff focused on the importance of making health outcome metrics available to patients:

*“We had this idea of having metrics that were readily available- the internal metrics, but also external metrics, depending on our website- measures for each of the Quadruple Aim … we haven’t settled on what we would publish and a system for maintaining that.” (ADMIN1)*

A unique element of MQGP is its proximity to specialist clinics and hospital facilities, allowing for patients to be referred to specialists on-site. As a private billing organisation, one doctor highlighted the need for transparency about out-of-pocket costs associated with patients being referred to specialists that operate adjacent to the general practice. Other doctors commented on the importance of patients knowing additional information about specialists, such as the days that they work and their subspecialties.

***“****It’d be useful to have an idea of out-of-pocket costs … to be able to give them some idea of what they might have to pay. It actually starts with us as well, the transparency about referrals.” (GP15)*

All of this suggests that MQGP broadly values transparency and has moved away from traditional approaches to funding and incentivising medical staff. Including both salaries and KPIs as financial incentives encourages not only value-based care, but also involvement in other research and teaching activities, which are important components of an LHS.

#### Continuous learning culture

Vital to the success of an LHS is the culture of learning; which is one supported by leaders within the organisation, and emphasises ongoing reflection and skill-building for staff [5]. The most frequently referenced aspect of the continuous learning culture by staff were the weekly update emails that were circulated by the business manager. These emails included updates on changes to health guidelines (particularly pertaining to COVID-19), invitations to educational events, and publicly acknowledged staff achievements. These updates were welcomed by clinical and non-clinical staff alike.

*“We get a newsletter every week from [ADMIN1] who is our manager, and he updates protocols on a weekly basis.” (ADMIN 12)*

The university environment was identified as a contributor to a culture of learning within the practice, as it presented frequent opportunities to engage in teaching, supervision, and learning:

*“That’s the other thing … if you’re teaching students you have to make sure that your knowledge is up to date as well, it’s inherent in this environment.” (GP7)*

*“They provide educational sessions, they have collaborative discussions with each other. I think a lot of us are involved with the university. They provide us with access to resources and we’ve got social media groups that we can work together to improve learning as well.” (GP2)*

One doctor highlighted the value of grand rounds at the adjacent hospital as both an opportunity to learn and meet specialists that worked in the adjacent clinics:

*“Grand rounds was probably the most powerful unifying meeting or unified one single point of contact for the whole of the clinic and it was very solidifying. Everyone was there once a month, chit chat beforehand, chit chat afterwards.” (GP6)*

The practice’s affiliation with an academic institution was the greatest contributor to the culture of learning, predominantly through opportunities for staff to be involved with teaching and supervision within the university. All-in-all, the value placed on learning and reflection by the leadership team, and the constant communication to staff, created an environment where staff were engaged in educational initiatives.

#### Structure and governance

‘Structure and governance’ were proposed in 2020 as an addition to the NAM’s framework for LHSs [5]. Governance structures can assist to facilitate progress toward an LHS by enabling policies and regulations that facilitate research, collaboration and learning. Participants were asked about what governance structures were in place to contribute to the learning culture in the practice. Such structures included multidisciplinary working groups for chronic disease management that involved both clinical and non-clinical staff, and a mentoring system between doctors, nurses, and administrative staff.

*“We have a doctor-buddy system as well. I’d be allocated to three or four different doctors, but then there’d be a few admin staff as well.” (ADMIN9)*

Participants were also asked about the nature of the collaborations for staff within and between the two practice sites, as well as with staff in the adjacent specialist and allied health clinics. Responses revealed that despite co-locating within one building with the specialist clinics, there were few opportunities to interact besides when referring patients.

*“I think there is a lot of collaboration between each [MQGP] clinic, it’s kind of odd though that there’s not a lot of collaboration between uni clinics. We are all sharing the same building, I always thought that that was kind of odd.” (ADMIN3)*

Whilst the practice did not have specific policies in place to facilitate learning and collaboration, they were implicit. The willingness of leadership to participate in research and QI initiatives enabled facilitation of several aspects of the LHS, further emphasising the crucial role of leadership in creating a culture of learning.

### Aim 2: outcomes from the embedded research

Embedding a researcher within a site of healthcare delivery has clearly articulated benefits in the research process by enhancing access and buy-in among participants and facilitating system learning [24]. Ideally, such a researcher should be co-located within the site, if only on a part time basis. This was the original plan in this study, however, two months after agreeing to include an embedded researcher, the outbreak of the Delta variant of COVID-19 resulted in a city-wide lockdown of Sydney, lasting 107 days, and limiting the prospect of physical co-location. Despite the impact of COVID-19, the embedded researcher coordinated regular monthly meetings of the project steering committee, and fortnightly meetings with the business manager and one academic GP. The researcher was also included in the practice emails and was able to access and use the CAT4 software to better understand the demographics of the practice population. These remote ways of working, ubiquitous in the COVID-19 period, still allowed for a degree of embedding to occur. From these strategies the researcher gained detailed insights into methods of staff communication, day-to-day clinic activities, acceptability of implemented initiatives (e.g., mobile phone app) and changes to practice operations aligning with changing COVID-19 guidelines. Despite the significant impact of COVID-19 on the healthcare system, the embedded research approach was facilitated in part through the engagement and commitment of the clinical and administrative practice staff, reflected by their regular attendance of steering committee meetings and ongoing communication with researchers.

The embedded researcher model offers broader benefits in exposing a health system workforce to empirical research, helping capacity-build research skills among the practice staff, and supporting longer-lasting and more meaningful partnerships between them and researchers, as opposed to transactional, project-based work [25]. A range of metrics point to success in this regard over the duration of the study: the project team expanded to include two additional GPs: one senior academic GP, and the other a recently trained GP. The Business Manager (FL) at MQGP took up an adjunct research role within the institute where the research was taking place, and one GP planned to undertake a PhD under the supervision of some members of the research team. The embedded researcher (GD) and senior research fellow (LAE) also joined another QI study with members of MQGP. The partnership between the practice and research institute leveraged further research opportunities, resulting in additional grant and funding applications that were led by clinic staff, and supported by the researchers.

## Discussion

This research is the first to present a case study of an LHS within an Australian primary care setting and one of the few to do so internationally. Interviews with practice staff indicated that MQGP is operating within several dimensions of the LHS framework, and there is a general willingness of staff to embrace additional elements of the LHS.

Science, informatics and technology are often the most discussed element of LHSs throughout the literature [8, 17, 18]. Some clear challenges pertain to the utility of technology and data as a tool for QI within MQGP. When interviewed, staff commented on the potential utility of the CAT4 technology whilst frequently highlighting the difficulty of finding the time to look at patient data, and many were not aware of the technology or its utility at all. This is a sentiment that has been discussed previously in relation to the barriers to LHS implementation [12]. Whilst solutions may include hiring additional staff to manage patient data and QI activities or ensuring that there is time set aside in clinicians’ days to focus on QI initiatives, this may require significant upfront and ongoing investment [26, 27] from the practice and a change in workplace culture. Such a cultural shift would expect clinicians to place value not only on treating individual patients, but also reviewing data at a broader practice-based level, potentially without remuneration. With access to huge volumes of information, a major challenge for those working in primary care settings is the ability to select the information that will be meaningful to clinicians and benefit the practice. EHRs have been touted as a central component of a functional LHS [28]. In 2012, the Australian Department of Health invested $2 billion for the development of ‘My Health Record’, an EHR system that allows patients and clinicians to access patient data. In 2020–2021, despite officially having 23 million active records, just over 10% of all registered users actually accessed their record [29], highlighting a potential missed opportunity for this tool to be utilised within a practice-level LHS.

Although the science and informatics domain is the most commonly discussed LHS feature [17, 18], the other domains, centred on continuous learning culture, patient clinician partnerships, and governance structures are not only crucial to a functioning LHS, but represent where MQGP excels as an LHS. Building stronger patient-clinician relationships and giving patients the opportunity to provide feedback to staff is a key element of MQGP’s functioning. Giving patients access to their medical records has been discussed as one way to engage and empower patients [30], and is something that would be supported by activities such as the implementation of the MyPractice app. Providing patients with the opportunity to contribute to the design and delivery of care has the benefits of improving services [31] and increasing patient confidence in the system.

The use of incentives within the practice was contentious. Despite KPIs existing for doctors, similar incentives were not available to administrative or nursing staff, contributing to a system in which some staff were financially incentivised to provide high-value care and improve the quality of the service, and some were not. Whilst all staff acknowledged that providing high quality care was their primary goal, it is crucial that ongoing discussions around incentives be inclusive and allow for all staff to benefit. As the practice continues to work towards embracing the LHS, incentives should be considered as a means to assist with implementation activities [4].

Participants commented on the appeal of working within a university environment, and how this facilitated ongoing teaching and learning opportunities, and a culture of continuous learning. Although not all primary care providers have this opportunity, our study highlights the benefit of cross-institutional partnerships, especially when these partnerships facilitate greater research opportunities for clinicians.

Based on the findings of this study, several recommendations for operationalising an LHS in primary care are listed (see Table 2). These recommendations are designed as a guide that can be used by practices in their journey toward an LHS but are not exhaustive, nor the only way to operationalise these domains.

**Table 2:** Recommendations for operationalising an LHS in primary care settings

Domain	Description	Recommendation
**Science and Informatics**	*Real time access to knowledge*	• Providing access to subscription based educational platforms. • Hosting lunchtime teaching sessions on topical health issues. • Upskilling clinicians to use clinical auditing tools to provide practitioners with overview of their patient cohort.
**Patient clinician partnerships**	*Digital capture of the care experience*	• Implementing technology which provides patients with access to referrals, prescriptions, certificates.
	*Engaged, empowered patients*	• Encouraging patients to leave online reviews and provide staff with feedback. • Holding focus groups with patients to discuss the implementation of new initiatives within the practice.
**Incentives**	*Incentives aligned for value*	• Paying staff a salary so that their remuneration is not based on care volume. • Creating KPIs and incentives that apply to all staff, not just doctors. • Creating financial incentives that are based not only on care delivered, but engaging in research, teaching, and supervision.
	*Full transparency*	• Publishing or making available metrics on patient health outcomes, linked to the quadruple aim.
**Culture**	*Leadership-instilled culture of learning*	• Creating affiliations with academic institutions, providing teaching, research and learning opportunities for staff.
	*Supportive system competencies*	• Holding regular meetings involving clinical and non-clinical staff that address quality improvement.
**Structure and governance**	*Policies, governance, and regulations aligned to facilitate research, collaboration, and learning*	• Forming multidisciplinary working groups that involve both clinical and non-clinical staff. • Encouraging senior leadership staff to engage with research opportunities and collaborations.

### Implications

**Fig. 1:**
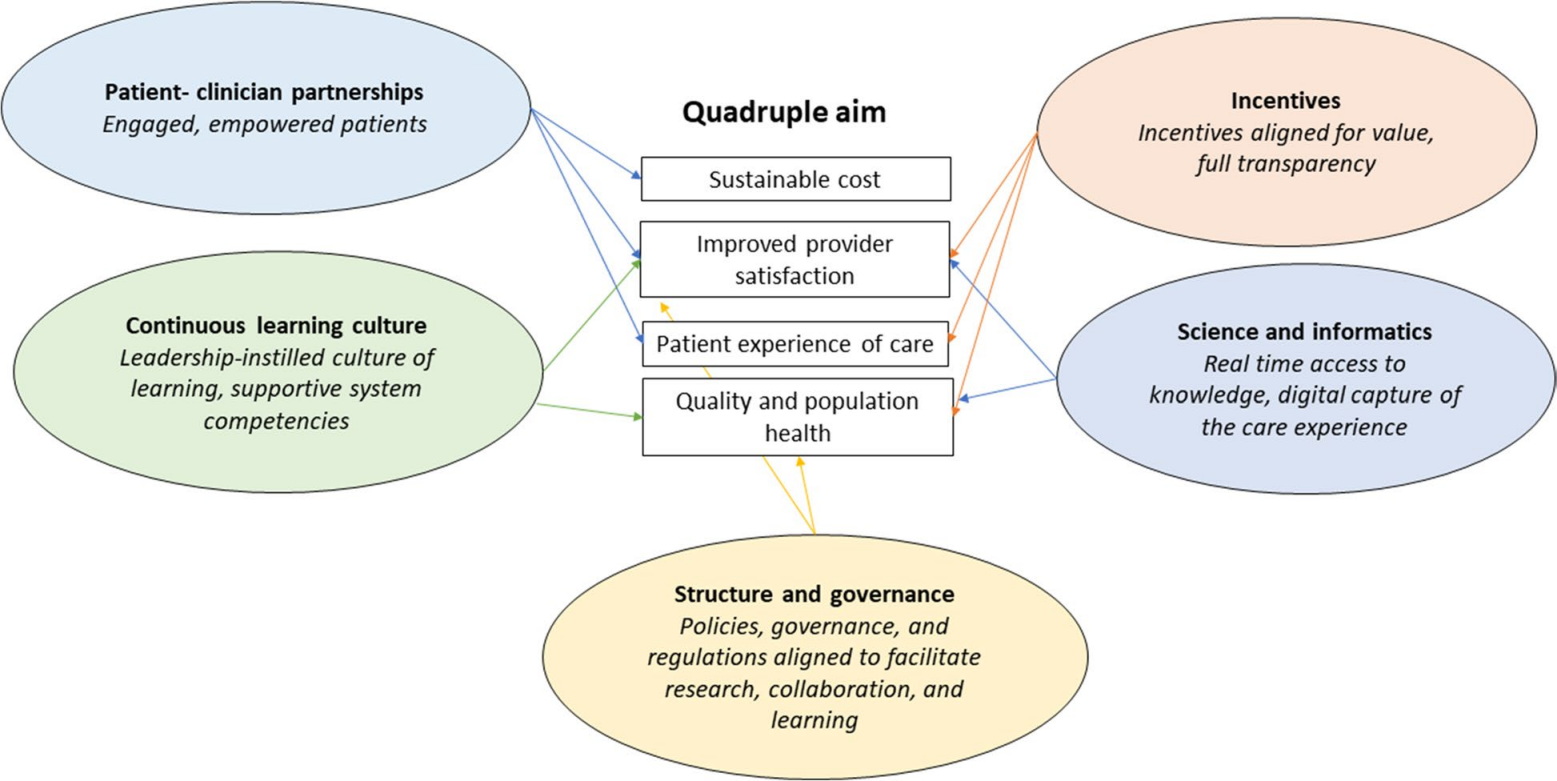
The relationship between the LHS framework and quadruple aim. Each arrow represents the way in which the LHS dimension enables aspects of the quadruple aim

In discussing QI in primary care, the quadruple aim [11] is often cited as being a central element of optimising system performance [11]. Thematic analysis of interview data showed that for many there was a considerable overlap between the dimensions of the LHS framework and the quadruple aim framework. It seems evident that adopting an LHS model will assist in making progress with the quadruple aim (see Fig. 1), resulting in the potential for improved performance of the healthcare system. The benefits of utilising an embedded researcher approach are noteworthy. In the present study, this approach facilitated regular contact between researchers and the stakeholders within the system and resulted in additional research projects emerging as an adjunct to the original research. Having an embedded researcher or researcher-in-residence [32] work across multiple projects within an organisation can bolster the researcher’s contextual knowledge and understanding of how the organisation operates, strengthen the relationships with staff working within the organisation, and can lead to opportunities for future collaboration.

### Strengths and limitations of this study

The utility of an embedded researcher to assist with implementation has been increasingly discussed in the literature [33–36]. The present study aimed to embed a researcher within the general practice prior to conducting interviews to better understand the context of how the practice operated, however co-location was limited due to COVID-19 lockdowns and restrictions. A strength of this study is the novel co-designed methodology, allowing research staff and practice staff to agree on how best to operationalise the LHS framework within a general practice setting. The high participation rate from interviewed staff was another strength of the study and indicated that findings were likely generalisable to the broader practice. The study was further enabled by strong engagement from the leadership team within the healthcare organisation. Semi-structured interviews permitted free discussion and open dialogue between the interviewer and interviewee. The primary limitation of this study is the inclusion of only one practice, restricting the generalisability to other primary care settings within Australia and internationally. The practice’s location – adjacent to a university – was a significant enabler for many of the LHS dimensions, however, is relatively unusual for a typical Australian general practice, and those of other countries, further limiting generalisability. Despite this, our findings show the benefit of building strong connections with academic institutions to facilitate LHS uptake, and therefore may serve as an exemplar for other primary care settings that wish to accelerate their LHS journey.

## Conclusion

In healthcare, the quadruple aim is widely accepted as an important model to support health system performance. This research presents a case study of an LHS in primary care, showing how the LHS serves as a tool to assist organisations in making progress toward fulfilling the quadruple aim, as well as presenting the utility of using an embedded research approach. Our findings show the potential for a collaborative, strategically focused organisation to operate as an LHS.

## Data Availability

The data generated and analysed during the current study are not publicly available to maintain the privacy of participants. Data is available from the corresponding author upon reasonable request.

## Abbreviations

COVID-19: Coronavirus disease of 2019
EHR: Electronic health record
KPI: Key performance indicator
LHS: Learning health system
MQGP: MQ Health General Practice
NAM: National Academy of Medicine
PHN: Primary Health Network
QI: Quality improvement
